# Uterine Hæmorrhage at the Menopause

**Published:** 1909-03-06

**Authors:** 


### Gynæcology.

### UTERINE HÆMORRHAGE AT THE MENOPAUSE.

It is an established belief that women are liable to unusual hæmorrhage of uterine origin at the menopause. It is not, however, clearly realised that the vast majority of these hæmorrhages owe their origin to some distinct pathological change in the uterus itself, and, though possibly made worse by the physiological changes due to the menopause, are not in themselves strictly caused by the latter. Most of the tragedies met with in gynæcological practice are the result of assigning "the change of life" as the cause of uterine hæmorrhage when in fact this is caused by a carcinoma of the uterus. Fortunately this error is most commonly made by women themselves or their friends; but the medical profession is not altogether blameless in this respect. It is quite common in hospital practice to meet with women who have inoperable carcinomata of the uterus, and have been under medical treatment for months without any examination. These, indeed, can only be called the tragedies of gynæcological practice: they arise chiefly because the menopause is still regarded as a fertile source of uterine bleeding.

How has this fetish arisen, and, if true, why should the menopause cause unusual bleeding? The belief has arisen from clinical observations that a certain number of women have excessive menstrual and occasional intermenstrual losses which suddenly cease, and consequently are looked upon as physiological. If these losses are caused by the menopause alone we can only explain them by supposing that some unusual stimulus of a nervous or biochemical nature sets up unusual congestion of the uterine capillaries and subsequent bleeding; at the other extreme of menstrual life excessive bleeding may be the result of an excessive stimulus alone, and in the absence of a better explanation and with our present knowledge this view is commonly accepted. It is also accepted that deficient or excessive ovarian activity may cause diminished or increased menstrual flow at other times than the extreme ends of menstrual life. Consequently we cannot absolutely state that excessive bleeding is never caused by the menopause alone; but we can say that excessive bleeding caused by the menopause alone is always a *menorrhagia*—that is, either a prolongation of a menstrual period or an excessive loss at a period, or both. It is almost if not quite absolutely certain that intermenstrual bleeding at the menopause is always due to a pathological change in the uterus. The fact that these bleedings sometimes undergo a spontaneous cure and suddenly cease, never to return, is no disproof of this. The reason is clear: many of the cases are due to endometritis, the evidence of which is thickening of the endometrium and elongation of the cavity of the uterus. Such uteri do not of necessity bleed except under the stimulus of menstruation, and where this stimulus has suddenly ceased the bleeding may cease too. It must be said, however, that these remarks apply to chronic hypertrophic glandular endometritis. We shall see later that "senile endometritis," which is commonly due to atrophy or sclerosis of the endometrium, often combined with infection, may cause bleeding long after the menopause is passed.

A further difficulty arises in these patients if we attempt to define the limits of the menopause. Here again the menopause fetish asserts itself, for it is not uncommon to meet with women of 40 who have excessive bleeding and who have been told that this is due to the change of life. Such patients may not be within five years of the real cessation of menstruation, and but rarely have any of the associated symptoms of the menopause. The commonest time for menstruation to cease is from the 47th to the 5[illegible] year, and before this period climacteric symptoms are not at all common. Before we look upon the menopause as even a predisposing factor we must assure ourselves that some of the associated symptoms, such as "hot flushes," headaches, nerve disturbances, dyspepsias, etc., are really present.

Thorough and immediate investigation, then, is our safeguard in dealing with any hæmorrhage in a patient who is suspected of approaching the menopause, just as much as it is at any other period of menstrual life. We cannot too often reiterate that there is no condition more worthy of immediate thorough investigation than uterine hæmorrhage, and that our hope of curing uterine cancer depends entirely on early recognition. The causes of hæmorrhage at the menopause are these: first and most commonly endometritis, glandular in type, and its usual hæmorrhage made worse by excessive ovarian stimulus. It may not cause any excessive bleeding until the unusual stimuli of the approaching menopause set up excessive uterine congestion. The bleeding may be intermenstrual as well as menstrual. Associated with endometritis, mucous polypi and fibro-myomata may be contributing factors. Displacements, especially retrodeviations, may conspire to increase congestion at this period, but also owe their effects largely, if not solely, to the endometritis which accompanies them. Secondly, carcinoma of the cervix and less frequently of the body of the uterus. Here the bleeding is practically always irregular, intermenstrual in type, and called forth by coitus, straining, exertion, etc. Thirdly, changes in the uterine vessels and musculature form a not inconsiderable proportion of the lesions. Arterio-sclerosis of the uterine vessels has been shown to be a fertile source of bleeding, and is more likely to act at the menopause than at other periods. The diagnosis of any of these conditions may be difficult or impossible without an exploratory curettage; and in doubtful conditions of the cervix, removal of a wedge of tissue for microscopic examination is the only means of diagnosing an early cancer.

If the case is one of pure menorrhagia a trial of drugs may be made for a limited time, for pure menorrhagia is not likely to be caused by cancer. Ergot in full doses is the chief drug of value, but has no effect in arterio-sclerosis, and not always any in endometritis. The opium derivative cotarnine is more likely to be of value in vascular or muscular degeneration, but cannot be relied upon. If an excessive stimulus is suspected these drugs may be combined with bromides or cannabis indica. In all cases of irregular bleeding where no cause can be found on examination by ordinary methods no time must be lost; the uterine cavity should be dilated, explored, and curetted. Any tissue removed should be submitted to an expert microscopist in gynæcological diseases. In spite of contrary statements, we affirm most emphatically that properly preserved curetted fragments present no difficulties whatever in diagnosis to an experienced gynæcological pathologist.

